# Thermodynamic Analysis of Group-III-Nitride Alloying with Yttrium by Hybrid Chemical Vapor Deposition

**DOI:** 10.3390/nano12224053

**Published:** 2022-11-17

**Authors:** Mina Moradnia, Sara Pouladi, Muhammad Aqib, Jae-Hyun Ryou

**Affiliations:** 1 Department of Mechanical Engineering, University of Houston, Houston, TX 77204, USA; 2 Texas Center for Superconductivity at UH (TcSUH), University of Houston, Houston, TX 77204, USA; 3 Advanced Manufacturing Institute (AMI), University of Houston, Houston, TX 77204, USA; 4 Department of Electrical and Computer Engineering, University of Houston, Houston, TX 77204, USA; 5 Materials Science and Engineering Program, University of Houston, Houston, TX 77204, USA

**Keywords:** alloying, piezoelectric, HybCVD, thermodynamic calculations, thin film

## Abstract

Group-IIIb-transition-metal-alloyed wurtzite Group-IIIa-nitride (IIIb-IIIa-N) thin films have higher piezoelectric characteristics than binary IIIa-N for a broad range of applications in photonic, electronic, sensing, and energy harvesting systems. We perform theoretical thermodynamic analysis for the deposition and epitaxial growth of Y-alloyed GaN and AlN films by a newly introduced growth technique of hybrid chemical vapor deposition (HybCVD), which can overcome the limitations of the conventional techniques. We investigate the equilibrium vapor pressures in the source zones to determine the dominant precursors of cations for the input of the mixing zone. Then, we study the driving force for the vapor-solid phase reactions of cation precursors in the growth zone to calculate the relationship between the solid composition of Y*_x_*Ga_1−*x*_N and Y*_x_*Al_1−*x*_N and the relative amount of input precursors (Y vs. GaCl and AlCl_3_) in different deposition conditions, such as temperature, V/III precursor input ratio, and H_2_/inert-gas mixture ratio in the carrier gas. The *x*_Y_ composition in YAlN changes nearly linearly with the input ratio of cation precursors regardless of the growth conditions. However, YGaN composition changes non-linearly and is also substantially affected by the conditions. The thermodynamic analysis provides insight into the chemistry involved in the epitaxial growth of IIIa-IIIb-N by the HybCVD, as well as the information for suitable growth conditions, which will guide the way for ongoing experimental efforts on the improvement of piezoelectricity of the lead-free piezoelectric materials.

## 1. Introduction

Group IIIa-N (III-N) materials, such as aluminum nitride (AlN) and gallium nitride (GaN) thin films, draw increasing attention in piezoelectric applications due to their exceptional properties of high-temperature stability, spontaneous electric polarization, low dielectric permittivity, high sound velocity, efficient transduction, and high stiffness [1,2,3]. However, the piezoelectric coefficients and the resulting electromechanical coupling factors (*k_t_*^2^) of III-N materials are relatively low compared to those of currently dominant piezoelectric materials, such as lead zirconate titanate (Pb[Zr*_x_*Ti_1−*x*_]O_3_, PZT) [4].

To address the challenge of relatively low piezoelectric coefficients, ternary Group-IIIa-IIIb-nitride (IIIa-IIIb-N) alloys were proposed. The piezoelectric strain constants *d*_33_ and *d*_31_ of Group-IIIa-N thin films can be significantly enhanced by the incorporation of Group-IIIb transition metals, such as scandium (Sc) and yttrium (Y), due to the reduction of elastic constants, along with an increase in piezoelectric stress constants (*e*_33_ and *e*_31_), hence the significant increase in *k_t_*^2^. The transition-metal-alloyed wurtzite AlN films were proven to increase their piezoelectric coefficients, while retaining most other beneficial material properties [5,6,7]. Furthermore, the substitution of the Group-IIIa atoms (Al or Ga) with a larger Group-IIIb atom (Sc or Y) in III-N increases their wurtzite internal parameter, *u* (the length of the metal-nitrogen bond parallel to the *c*-axis relative to the lattice parameter), which can cause a local distortion in the wurtzite structure toward a layered-hexagonal structure and changes the tensile strain [8]. The IIIa-IIIb-N thin films can function as a ferroelectric material, as well as piezoelectric material, due to the presence of a transition metal which causes the induced strain. This strain sufficiently reduces the energy barrier between two polarization states of the III-N wurtzite structure for the ferroelectric polarization switching [9,10]. In addition, YGaN can be an alternative material for the active region of photon emitters with bandgap energies of 0.85–3.4 eV [11]. Higher piezoelectric coefficients of IIIa-IIIb-N alloys can extend the applications of the piezoelectric devices in wireless communication [12], sensors [13,14], mechanical energy harvesting [15,16], semiconductor-based ferroelectrics [10], and optoelectronics [17].

The growth of crystalline IIIa-IIIb-N thin films can be accomplished by different deposition techniques. However, each technique shows drawbacks: compromised crystalline quality and film uniformity control [18,19], even for the favorable magnetron sputtering growth technique for these metastable materials [20]; low volatility of the metalorganic precursors of transition metals [9,19] for metalorganic chemical vapor deposition (MOCVD) [21]; the requirement of an extremely high vacuum in a growth chamber and very low growth rates [22,23] for molecular beam epitaxy (MBE) [24]; and no chemical reaction for transition-metal-chloride to form their nitride for hydride vapor-phase epitaxy (HVPE) [25]. To address the econo-technical challenges of the existing growth techniques, we proposed a new growth technique, hybrid chemical vapor deposition (HybCVD), utilizing elemental sources, chloride and hydride, as precursors of the Group-IIIb transition-metal element, Group-IIIa element, and nitrogen, respectively, for the epitaxial growth of ScAlN films [26].

This new technique can be further extended to Y-alloyed IIIa-N films with additional advantages. First, an ab-initio calculation estimates the significant enhancement in the piezoelectric strain constant (*d*_33_) by increasing Y content. The *d*_33_ increases by 700% for wurtzite Y*_x_*Al_1-*x*_N, which is higher than that for Sc*_x_*Al_1-*x*_N (by 500%) [6]. Therefore, AlN alloying with Y could be better for the improvement of the piezoelectric properties. The piezoelectric coefficients of Y*_x_*Al_1−*x*_N (*x*_Y_ = 0.375) are found to be *d*_33_ = 17.5 pC/N, *d*_15_ = −11.07 pC/N, and *d*_31_ = −8.65 pC/N, which are ~300%, ~400%, and ~370% higher than those of AlN, respectively. The dielectric constants (*ε*_11_, *ε*_33_) slightly increase from (3.7, 5.1) for AlN to (5.3, 5.7) for Y_0.375_Al _0.625_N. Therefore, *k_t_*^2^ of YAlN (0 ≤ *x* ≤ 0.375) can be improved up to ~650% in relation to that of AlN [27]. Second, Y element is cheaper than Sc, which can lower overall material and manufacturing costs. Third, Y-alloyed IIIa-N can be better benefited by the HybCVD. GaN deposition by DC sputtering is very challenging due to the very low melting temperature of Ga (~86 °F). The lack of a highly volatile precursor of Y poses a challenge in the MOCVD growth of YAlN and YGaN alloys. Also, clustering was reported in the MOCVD growth by increasing Y vapor pressure in an attempt to achieve higher mole fractions of YN in the alloy. Whereas Leone et al. were able to grow a ScAlN alloy by employing several modifications to the standard MOCVD growth [17], the MOCVD growth of the YGaN alloy has not been reported.

In the present work, we theoretically study the thermodynamics of (1) precursor reaction chemistry and (2) solid-phase formation of YGaN and YAlN by the HybCVD with various deposition parameters, including growth temperatures, input V/III ratios, and input carrier gas ratios in different parts of source zones and mixing/growth zones.

## 2. Materials and Methods

### 2.1. Group-IIIa (Ga) Source-Zone Calculation

Ga-chloride vapor phases are formed in the source zone of Ga by the reaction between elemental Ga and HCl to estimate the amounts of precursors for Ga, which is the major cation content in the YGaN ternary phase. The equilibrium partial pressures of the chloride species in the source zone are used for the input of Ga precursors in the growth zone. Also, it is often important to control the relative amounts of chloride species to minimize their attack on quartz (SiO_2_), which is a typical material for the growth chamber. With input gases of HCl (reactant), H_2_, and inert gas (IG) (for carrier gas), seven gaseous species exist over the Ga metal (HCl, GaCl_3_, GaCl_2_, GaCl, Ga_2_Cl_6_, H_2_, and IG), and their reactions include:

Ga (s,l) + 3HCl (g) → GaCl_3_ (g) + 3/2H_2_ (g) (1)

Ga (s,l) + 2HCl (g) → GaCl_2_ (g) + H_2_ (g) (2)

Ga (s,l) + HCl (g) → GaCl (g)+ 1/2H_2_ (g) (3)

GaCl_3_ (g) + GaCl_3_ (g) → Ga_2_Cl_6_ (g) (4)

Equilibrium constants of the above reactions (Equations (1)–(4)) are:

(5) $$K_{1}=\frac{p_{{\mathrm{GaCl}}_{3}}\;p_{H_{2}}^{3/2}}{P_{\mathrm{HCl}}^{3}}$$

(6) $$K_{2}=\frac{p_{{\mathrm{GaCl}}_{2}}\;p_{H_{2}}}{p_{\mathrm{HCl}}^{2}}$$

(7) $$K_{3}=\frac{p_{\mathrm{GaCl}}\;p_{H_{2}}^{1/2}}{p_{\mathrm{HCl}}^{}}$$

(8) $$K_{4}=\frac{p_{{\mathrm{Ga}}_{2}{\mathrm{Cl}}_{6}}^{}}{p_{{\mathrm{GaCl}}_{3}}^{2}}$$

where *p_i_* is the equilibrium partial pressure of each gas in the reaction. The temperature-dependent *K_i_* values can be determined using NIST-JANAF thermochemical tables and HSC Chemistry software version 6 [28,29,30]. The total pressure of the source zone, *P*_total_, the sum of seven equilibrium partial pressures is:

(9) $$P_{\mathrm{total}}=\sum p_{i}{=p}_{\mathrm{GaCl}}{+p}_{{\mathrm{GaCl}}_{2}}{+p}_{{\mathrm{GaCl}}_{3}}{+p}_{{\mathrm{Ga}}_{2}{\mathrm{Cl}}_{6}}{+p}_{\mathrm{HCl}}{+p}_{H_{2}}{+p}_{\mathrm{IG}}$$

The input partial pressure, $p_{i}^{{}^{\circ}}$, of three input gases—HCl, H_2_, and IG—are related to a ratio of the number of chlorine atoms $\left(\frac{1}{2}p_{\mathrm{HCl}}^{{}^{\circ}}\right)$ to the number of hydrogen plus inert gas atoms $\left(\frac{1}{2}p_{\mathrm{HCl}}^{\circ }{+p}_{H_{2}}^{\circ }{+p}_{\mathrm{IG}}^{\circ }\right)$ and a ratio of the number of hydrogen atoms $\left(\frac{1}{2}p_{\mathrm{HCl}}^{\circ }{+p}_{H_{2}}^{\circ }\right)$ to the number of hydrogen plus inert gas atoms $\left(\frac{1}{2}p_{\mathrm{HCl}}^{\circ }{+p}_{H_{2}}^{\circ }{+p}_{\mathrm{IG}}^{\circ }\right)$ in the source-zone system, which is defined by *C* and *H* in the equations as follow:

(10) $$C=\frac{\frac{1}{2}{\;p}_{\mathrm{HCl}}^{\circ }}{\frac{1}{2}{\;p}_{\mathrm{HCl}}^{\circ }{+p}_{H_{2}}^{\circ }{+p}_{\mathrm{IG}}^{\circ }}$$

(11) $$H=\frac{\frac{1}{2}\;p_{\mathrm{HCl}}^{\circ }{+p}_{H_{2}}^{\circ }}{\frac{1}{2}\;p_{\mathrm{HCl}}^{\circ }{+p}_{H_{2}}^{\circ }{+p}_{\mathrm{IG}}^{\circ }}$$

Considering the mass conservation, the *C* and *H* do not change as the input number of each atom should be equal to the number of each atom in equilibrium partial pressures of the source zone:

(12) $$C=\frac{\frac{3}{2}\;p_{{\mathrm{GaCl}}_{3}}{+p}_{{\mathrm{GaCl}}_{2}}+\frac{1}{2}\;p_{\mathrm{GaCl}}{+3\;p}_{{\mathrm{Ga}}_{2}{\mathrm{Cl}}_{6}}^{}+\frac{1}{2}\;p_{{\mathrm{HCl}}_{}}}{\frac{1}{2}{\;p}_{\mathrm{HCl}}^{}{+p}_{H_{2}}^{}{+p}_{\mathrm{IG}}^{}}$$

(13) $$H=\frac{\frac{1}{2}\;p_{\mathrm{HCl}}{+p}_{H_{2}}}{\frac{1}{2}{\;p}_{\mathrm{HCl}}^{}{+p}_{H_{2}}^{}{+p}_{\mathrm{IG}}^{}}$$

The equilibrium partial pressures of gaseous phases (*p_i_*) can be calculated from Equations (5)–(13). The calculation is based on the definition of the input parameters: temperature (*T*), total equilibrium partial pressure (*P*_total_), input partial pressure of HCl ($p_{\mathrm{HCl}}^{{}^{\circ}}$), and input ratio of hydrogen in the carrier gas. The amounts of Ga precursors input are further controlled by adjusting the volume flow rate of total input gases in the Ga source zone.

### 2.2. Group-IIIa (Al) Source-Zone Calculation

We consider the same definitions and calculations for Al source zone for the growth of YAlN ternary phase, which is described in the earlier report [26]. The major difference between Ga and Al source zones is different chloride precursors as a dominant product carried to the mixing zone. The major chloride product in the Ga source zone is GaCl. However, AlCl_3_ should be the main precursor considering system integrity and growth-zone thermodynamics.

### 2.3. Group-IIIb Transition-Metal (Y) Source-Zone Calculation

Transition-metal-chloride precursors cannot be used for the deposition of transition-metal-alloyed III-N. The Y-chloride precursors can be produced by the following chemical reactions:

Y (s,l) + HCl (g) → YCl + 1/2H_2_ (g) (14)

Y (s,l) + 2HCl (g) → YCl_2_ (g) + H_2_ (g) (15)

Y (s,l) + 3HCl (g) → YCl_3_ (g) + 3/2H_2_ (g) (16)

From the Gibbs free energy changes for the chemical reactions in Equations (14)–(16) (Figure S1), the formation of YCl and YCl_2_ (Equations (14) and (15)) are not possible in a typical temperature range of the source zone. Only YCl_3_ can be produced. However, the Gibbs free energy for the formation of solid YN by the reaction between YCl_3_ and NH_3_ is positive at typical deposition temperatures (Figure S2):

YCl_3_ (g) + NH_3_ (g) → YN (s) + 3HCl (g) (17)

It is necessary to find an alternative precursor.

The elemental precursor of the transition metal can be provided from the equilibrium vapor phase over its condensed phase. For the Y case, the equilibrium vapor pressure can be controlled in a wide range with the exponential dependence of the vapor pressure by temperature and the high boiling point of the transition metals (Figure S3). When the vapor phase element can be transferred by carrier gases from the source zone to the mixing zone, the amount of Y input precursor in the mixing zone is also controlled by the volume flow rates of carrier gases over the transition metal source.

### 2.4. Growth-Zone Calculation

The obtained equilibrium partial pressures from each source zone become the input partial pressures in the growth zone. GaCl_2_, GaCl_3_, and Ga_2_Cl_6_ are ignored in the input gases in the growth zone of YGaN due to their extremely low equilibrium vapor pressures from Ga source-zone calculation (Figure S4 and Section 3.1.1). Therefore, 7 gaseous species of Y, GaCl, NH_3_, HCl, H_2_, IG, and GaCl_3_ (as a by-product) are considered. Three possible reactions of these species include:

Y (g) + NH_3_ (g) → YN (s, alloy) + 3/2H_2_ (g) (18)

GaCl (g) + NH_3_ (g) → GaN (s, alloy) + HCl (g) + H_2_ (g) (19)

GaCl (g) + 2HCl (g) → GaCl_3_ (g) + H_2_ (g) (20)

Equilibrium constants corresponding to the above reactions of Equations (18) to (20) are:

(21) $$K_{5}=\frac{a_{\mathrm{YN}}\;p_{H_{2}}^{3/2}}{p_{Y}{\;p}_{{\mathrm{NH}}_{3}}}$$

(22) $$K_{6}=\frac{a_{\mathrm{GaN}}\;p_{\mathrm{HCl}\;}^{}p_{H_{2}}}{p_{\mathrm{GaCl}}\;p_{{\mathrm{NH}}_{3}}}$$

(23) $$K_{7}=\frac{p_{H_{2}}\;p_{{\mathrm{GaCl}}_{3}}}{p_{\mathrm{GaCl}}{\;p}_{\mathrm{HCl}\;}^{2}}$$

where *p_i_* is the equilibrium partial pressure of each gas in the reaction and *a*_YN_ and *a*_GaN_ are the activities of the binary compounds in the YGaN alloy. The interaction parameter between wurtzite YN and wurtzite GaN, Ω_YN(wurtzite)-GaN(wurtzite)_ for the calculation of activities based on a regular solution model, is estimated to be 55,853 cal/mol [31]. Total pressure of the growth zone, *P*_total_, is the sum of equilibrium partial pressures of 7 gases:

(24) $$p_{\mathrm{total}}=\sum p_{i}{=p}_{Y}{+p}_{\mathrm{GaCl}}{+p}_{{\mathrm{GaCl}}_{3}}{+p}_{{\mathrm{NH}}_{3}}{+p}_{\mathrm{HCl}}{+p}_{H_{2}}{+p}_{\mathrm{IG}}$$

Considering the stoichiometric relationship between cation (Y and Ga) and anion (N) in Y*_x_*Ga_1-*x*_N formation, the solid phase amount of Y and Ga should be the same as that of N. According to the mass conservation law, the amount of each element in the solid phase is the difference between input partial pressure (*p_i_*^°^) and equilibrium partial pressures (*p_i_*) associated with the element. The amount of Y and Ga in solid is:

(25) $$p_{\mathrm{GaCl}}^{{}^{\circ}}{+p}_{Y}^{{}^{\circ}}-{(p}_{\mathrm{GaCl}}{+p}_{{\mathrm{GaCl}}_{3}}{+p}_{Y})$$

The amount of N in solid ${(p}_{{\mathrm{NH}}_{3}}^{{}^{\circ}}-p_{{\mathrm{NH}}_{3}}$) is the same as Equation (25) by the stoichiometry:

(26) $$p_{\mathrm{GaCl}}^{{}^{\circ}}{+p}_{Y}^{{}^{\circ}}-{(p}_{\mathrm{GaCl}}{+p}_{{\mathrm{GaCl}}_{3}}{+p}_{Y}{)=p}_{{\mathrm{NH}}_{3}}^{{}^{\circ}}-p_{{\mathrm{NH}}_{3}}$$

Again, the number of Cl and H in input gases should be the same as the number of each atom in equilibrium by mass conservation:

(27) $$C=\frac{\frac{1}{2}\;p_{\mathrm{GaCl}}^{{}^{\circ}}}{\frac{3}{2}{\;p}_{{\mathrm{NH}}_{3}}^{{}^{\circ}}{+p}_{H_{2}}^{{}^{\circ}}{+p}_{\mathrm{IG}}^{{}^{\circ}}}=\frac{\frac{1}{2}\;p_{\mathrm{GaCl}}+\frac{3}{2}{\;p}_{{\mathrm{GaCl}}_{3}}+\frac{1}{2}{\;p}_{{\mathrm{HCl}}_{}}}{\frac{3}{2}{\;p}_{{\mathrm{NH}}_{3}}^{}+\frac{1}{2}\;p_{\mathrm{HCl}}{+p}_{H_{2}}^{}{+p}_{\mathrm{IG}}^{}}$$

(28) $$H=\frac{\frac{3}{2}\;p_{{\mathrm{NH}}_{3}}^{{}^{\circ}}{+p}_{H_{2}}^{{}^{\circ}}}{\frac{3}{2}{\;p}_{{\mathrm{NH}}_{3}}^{{}^{\circ}}{+p}_{H_{2}}^{{}^{\circ}}{+p}_{\mathrm{IG}}^{{}^{\circ}}}=\frac{\frac{3}{2}\;p_{{\mathrm{NH}}_{3}}^{}+\frac{1}{2}\;p_{\mathrm{HCl}}{+p}_{H_{2}}}{\frac{3}{2}{\;p}_{{\mathrm{NH}}_{3}}^{}+\frac{1}{2}\;p_{\mathrm{HCl}}^{}{+p}_{H_{2}}^{}{+p}_{\mathrm{IG}}^{}}$$

We consider 5 input gases, including GaCl, Y, H_2_, IG (carrier gases), and NH_3_, excluding GaCl_2_ and GaCl_3_ from the Ga source zone for the reason explained earlier. The reaction condition is defined by deposition temperature (*T*), total pressure (*P*_total_), input partial pressure of GaCl ($p_{\mathrm{GaCl}}^{{}^{\circ}}$), input V/III ratio, and the mixing of hydrogen in the carrier gas. Then, from Equations (21)–(28), equilibrium partial pressures of vapor phases are calculated to estimate the Y composition in the alloy compound of Y*_x_*Ga_1−*x*_N.

A similar approach is used for YAlN alloys. However, due to the difference in the dominant Group-IIIa precursor (GaCl vs. AlCl_3_), different possible reactions are considered for 6 gaseous species of Y, AlCl_3_, NH_3_, HCl, H_2_, and IG.

Two possible reactions of these species include:

Y (g) + NH_3_ (g) → YN (s, alloy) + 3/2H_2_ (g) (29)

AlCl_3_ (g) + NH_3_ (g) → AlN (s, alloy) + 3HCl (g) (30)

Equilibrium constants corresponding to the above reactions of Equations (29) and (30) are:

(31) $$K_{8}=\frac{a_{\mathrm{YN}}\;p_{H_{2}}^{3/2}}{p_{Y}\;p_{{\mathrm{NH}}_{3}}}$$

(32) $$K_{9}=\frac{a_{\mathrm{AlN}}\;p_{\mathrm{HCl}\;}^{3}}{p_{{\mathrm{AlCl}}_{3}}\;p_{{\mathrm{NH}}_{3}}}$$

where *a*_AlN_ represents the activities of the binary compounds in the YAlN alloy. The interaction parameter between wurtzite YN and wurtzite AlN, Ω_YN(wurtzite)-AlN(wurtzite)_, is estimated to be 77,361 cal/mol. We do not include the formation of Al_2_Cl_6_ in our calculation due to its positive Gibbs free energy in typical growth zone temperatures (Figure S5).

## 3. Results

### 3.1. Input Precursor Control

Thermodynamic analysis in the source zone provides information on the amount of partial pressure of vapor species in the form of chloride or elemental source for the input of the mixing/growth zone.

#### 3.1.1. Precursor from Ga Source Zone

To evaluate and control the amounts of Ga precursors, the equilibrium partial pressures of reactants and products in the Ga source zone as a function of temperature at a fixed total pressure, *P_total_* = 1 atm (no vacuum is necessary), are calculated. The partial pressures of other input gases are $p_{\mathrm{HCl}}^{{}^{\circ}}$ = 0.00008 atm and $p_{H_{2}}^{{}^{\circ}}$ = 0.0999 atm with remaining IG. Reactions between Ga and HCl form GaCl, GaCl_2_, GaCl_3_, and Ga_2_Cl_6_. At all the temperature ranges (up to 900 °C) of Ga source zone, GaCl is a dominant species among the chlorides (Figure S4). For instance, at a source-zone temperature of 500 °C, the equilibrium partial pressure of GaCl is significantly higher than those of GaCl_2_, GaCl_3_, and Ga_2_Cl_6_ by the order of 10 in magnitude, i.e., 10^−5^ atm ($p_{\mathrm{GaCl}}$) vs. 10^−14^ atm ($p_{{\mathrm{GaCl}}_{2}}$, $p_{{\mathrm{GaCl}}_{3}}$, and $p_{{\mathrm{Ga}}_{2}{\mathrm{Cl}}_{6}}$). Hence, only GaCl is considered as a precursor of Ga in the growth zone.

For the reaction between Ga chlorides and the chamber wall, the change in the equilibrium constant, *K_i_*, as a function of reciprocal temperature, is negative for all the reactions between different chlorides of Ga and SiO_2_ (Figure S6); therefore, there is no concern for the degradation of the chamber wall/liner made of quartz. This is different from the Al source zone, where the accurate control of source-zone temperature is necessary to protect the chamber walls. The temperature of the Al source zone should be maintained lower than ~550 °C to dominantly produce the AlCl_3_ and suppress the formation of AlCl_2_, AlCl, and Al_2_Cl_6_ that react with the chamber liner and walls. The calculated results provide a condition for introducing GaCl as a dominant Ga chloride in the Ga source zone in order to transfer the Group-III element to the mixing/growth zone by the assistance of the carrier gases. The thermodynamic calculation of Al source zone was described in detail in the previous paper on the HybCVD of ScAlN [26].

#### 3.1.2. Precursor from Y Source Zone

A high vapor pressure can be achieved in the transition-metal source (Y) zone (Figure S3), since the boiling temperature of Y is very high at ~3338 °C. However, the source-zone temperature is not too high, and it is preferably lower than or similar to the growth-zone temperature. At temperature of ~1200 °C, the Y equilibrium vapor pressure is 1.95 × 10^−8^, atm which is significantly lower than that of GaCl, e.g., ~10^−4^ atm at most temperatures (Figure S4). A higher flow rates of carrier gas in the Y source zone may compensate this lower Y equilibrium vapor pressure. However, the difference is too much to be compensated by the flow rate control. Nevertheless, low vapor pressure of Y does not cause a serious issue in the growth of YGaN, which will be discussed in Section 4.2.

The Y reactivity with the source zone components should be considered in their materials selection. Pyrolytic boron nitride (BN), graphite, silicon-carbide (SiC)-coated graphite, and alumina (Al_2_O_3_) are made of materials that can be used for the Y storage container. Based on thermodynamic calculations of the Gibbs free energy changes of Y reaction with various materials for the container, the best way to prevent any damage to the container is to make the container from SiC-coated graphite or graphite (Figure S7).

### 3.2. Alloy Composition Control

Previous studies show that the high-quality crystalline structure of YAlN can be achieved at Y composition below ~25% where the complete mixing of YN and AlN in wurtzite structure is stable as the crystallinity degrades in Y*_x_*Al_1−*x*_N at higher *x*_Y_ [32]. Therefore, in the present study for the thermodynamic calculation of Y*_x_*Ga_1−*x*_N and Y*_x_*Al_1−*x*_N thin-film growth, only the conditions that can result in piezoelectric films with lower *x*_Y_ are considered. The amount of input Y precursor with respect to the total amount of input cation precursors, input Y ratio, is defined as one of follows (Equation (33) for YGaN and Equation (34) for YAlN):

(33) $$R_{Y}=\frac{p_{Y}^{{}^{\circ}}}{p_{\mathrm{GaCl}}^{{}^{\circ}}{+p}_{Y}^{{}^{\circ}}}$$

(34) $$R_{Y}=\frac{p_{Y}^{{}^{\circ}}}{p_{{\mathrm{AlCl}}_{3}}^{{}^{\circ}}{+p}_{Y}^{{}^{\circ}}}$$

The range of *R*_Y_ is limited up to ~0.3 in the calculation of the relationship between the cation precursor input ratios (*R*_Y_) and solid compositions (*x*_Y_) (i.e., the mole fraction of YN in Y*_x_*Ga_1−*x*_N or Y*_x_*Al_1−*x*_N), considering the range maintaining the piezoelectric properties of the deposited film.

Alloy composition *x*_Y_ in Y*_x_*Ga_1−*x*_N (or Y*_x_*Al_1−*x*_N) thin films is studied by changing the relative input amounts of Y and Ga (or Al) precursors. At various *R*_Y_, equilibrium partial vapor pressures of reactants and products are calculated at selected temperatures (*T_g_*), input ratios of Group-V precursor to Group-III precursors including both IIIa and IIIb precursors (V/III ratios), and input carrier-gas mixture ratios (effect of H_2_ in the carrier gas). The driving force for the deposition of transitional metal III-N alloy is the difference between input partial pressure ($p_{i}^{{}^{\circ}}$) and equilibrium partial pressure ($p_{i}$) of cation precursors, corresponding to the amount consumed in the vapor-solid reaction. The difference in each cation precursor, i.e., $p_{Y}^{{}^{\circ}}-p_{Y}$ and $p_{\mathrm{GaCl}}^{{}^{\circ}}-p_{\mathrm{GaCl}}-p_{{\mathrm{GaCl}}_{3}}$, determines the relative ratio of Y and Ga in the deposited alloy film. Therefore, the solid composition of Y, *x*_Y_ in Y*_x_*Ga_1−*x*_N film is defined by the following equation:

(35) $$x_{Y}=\frac{p_{Y}^{{}^{\circ}}-p_{Y}}{\left(p_{Y}^{{}^{\circ}}-p_{Y}\right)+\left(p_{\mathrm{GaCl}}^{{}^{\circ}}-p_{\mathrm{GaCl}}-p_{{\mathrm{GaCl}}_{3}}\right)}$$

A carrier gas is required to transfer the vapor-phase precursors from the source zones into the mixing and growth zones. This carrier gas should be preferably an inert gas (IG), such as argon (Ar) and/or hydrogen (H_2_). While nitrogen (N_2_) gas is also a common carrier in CVDs, it should be avoided for Y precursors due to its high tendency to react with Y.

#### 3.2.1. Temperature Effect on YGaN Deposition

**Figure 1:**
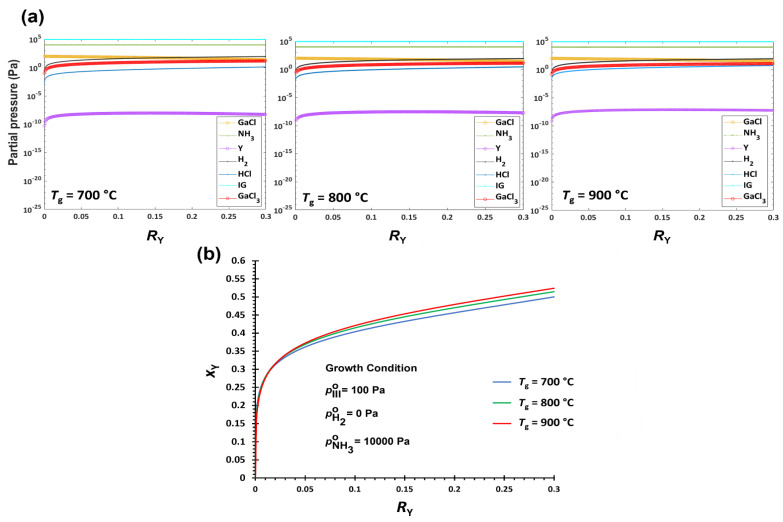
(**a**). Equilibrium partial pressures of carrier gas, reactants, and products in the growth zone of YGaN. (**b**). Mole fraction of YN, *x*_Y_, in the deposited Y*_x_*Ga_1−*x*_N solid film as a function of cation precursor input ratio, *R_Y_*, at various growth temperatures, *T_g_*.

Temperature is the most critical growth parameter in the CVD method. Figure 1a shows the changes in the equilibrium partial pressures of $p_{\mathrm{GaCl}}$, $p_{{\mathrm{GaCl}}_{3}}$, $p_{{\mathrm{NH}}_{3}}$, $p_{Y}$, $p_{H_{2}}$, $p_{\mathrm{HCl}}$, and $p_{\mathrm{IG}}$ as a function of *R*_Y_ at various *T_g_* (700–900 °C). The other growth conditions are fixed at $p_{H_{2}}^{\circ }$ = 0 Pa (no H_2_ in carrier gas), $p_{\mathrm{HCl}}^{\circ }$ = 0.1 Pa, and $p_{{\mathrm{NH}}_{3}}^{\circ }$ = 10,000 Pa. Input partial pressures of the Y precursor (Y), $p_{Y}^{\circ }$, and the Ga precursor (GaCl), $p_{\mathrm{GaCl}}^{{}^{\circ}}$, are varied at the fixed total input partial pressures of cation (Y and Ga) precursors, $p_{Y}^{\circ }{+p}_{\mathrm{GaCl}}^{{}^{\circ}}$ = $p_{\mathrm{III}}^{\circ }\;$= 100 Pa. Hence, the V/III ratio is also fixed at 100. The equilibrium partial pressure of Y, $p_{Y}$, is lower than that of GaCl, $p_{\mathrm{GaCl}}$, by several orders of magnitude in all the given conditions (Figure 1a). A significantly higher equilibrium constant, *K*_5_, in Equation (21) than *K*_6_ in Equation (22) is in line with this difference. This behavior indicates a higher driving force for YN formation than that of GaN in given conditions. Both the amounts of $p_{Y}$ and $p_{\mathrm{GaCl}}$ increase with an increase in *T_g_*, i.e., the same trend of driving force with temperature. However, the increments in $p_{\mathrm{GaCl}}$ with higher temperatures are significantly higher than those in $p_{Y}$. While the amount of $p_{{\mathrm{GaCl}}_{3}}$ decreases with increasing *T_g_*, the change is marginal as compared to the change in $p_{\mathrm{GaCl}}$. Hence, it cannot make a substantial difference in the driving force of GaN formation. At higher temperatures, therefore, the GaN formation becomes less efficient. Consequently, *x*_Y_ increases with temperature at the same *R*_Y_. Also, the difference is more recognizable with higher *R*_Y_, as shown in Figure 1b. Furthermore, slower growth rates are expected at higher temperatures due to slightly reduced driving forces for both the YN and GaN (Figure S5).

#### 3.2.2. Hydrogen Effect on YGaN Deposition

**Figure 2:**
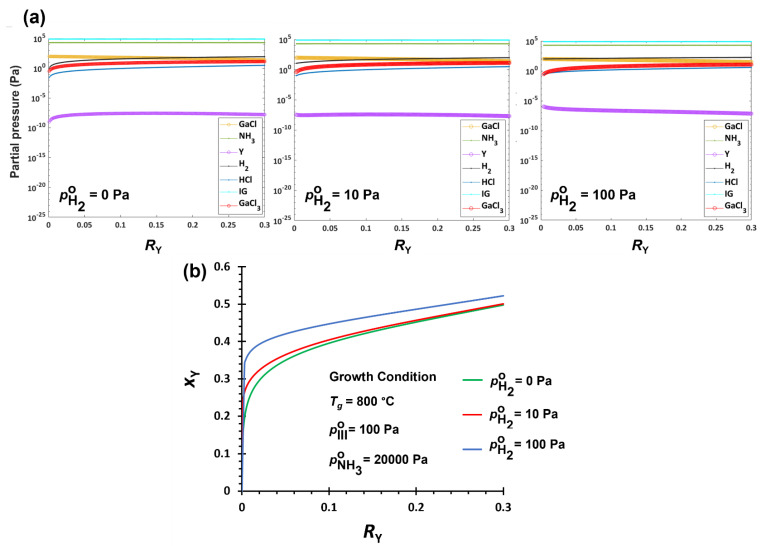
(**a**). Equilibrium partial pressures of carrier gas, reactants, and products in the growth zone of YGaN. (**b**). Mole fraction of YN, *x*_Y_ in the deposited Y*_x_*Ga_1−*x*_N solid film as a function of cation precursor input ratio, R_Y_, with different H_2_ input conditions.

The effect of the input mixture of carrier gases is another factor to be considered. IG and H_2_ are used as carrier gases in conventional CVD processes, and their mixture ratio can affect the deposition of YGaN by participation of H_2_ in the chemical reactions. Especially, H_2_ is the by-product of possible reactions for the formation of YN and GaN (Equations (18)–(20)). Figure 2a shows the changes in equilibrium partial pressures of $p_{\mathrm{GaCl}}$, $p_{{\mathrm{GaCl}}_{3}}$, $p_{{\mathrm{NH}}_{3}}$, $p_{Y}$, $p_{H_{2}}$, $p_{\mathrm{HCl}}$, and $p_{\mathrm{IG}}$ as a function of *R*_Y_ at the H_2_ input range of $p_{H_{2}}^{\circ }$ = 0–100 Pa. The other conditions are fixed at growth temperature *T_g_* = 800 °C, NH_3_ input partial pressure $p_{{\mathrm{NH}}_{3}}^{\circ }$ = 20,000 Pa, and $p_{Y}^{\circ }{+p}_{\mathrm{GaCl}}^{{}^{\circ}}$ = $p_{\mathrm{III}}^{\circ }$ = 100 Pa. Hence, the V/III ratio is $p_{{\mathrm{NH}}_{3}}^{\circ }$/($p_{Y}^{\circ }{+p}_{\mathrm{GaCl}}^{{}^{\circ}}$) = 200. When comparing $p_{Y}$ and $p_{\mathrm{GaCl}}$ at varied $p_{H_{2}}^{\circ }$, both $p_{\mathrm{GaCl}}$ and $p_{Y}$ increase with higher $p_{H_{2}}^{\circ }$, while $p_{{\mathrm{GaCl}}_{3}}$ decreases. Variation of $p_{Y}$ is marginal compared to the change of $p_{\mathrm{GaCl}}$ and $p_{{\mathrm{GaCl}}_{3}}$. The effect of higher $p_{\mathrm{GaCl}}$ on the driving force of GaN formation is more than that of lower $p_{{\mathrm{GaCl}}_{3}}$, resulting in lower GaN formation. The increments in $p_{\mathrm{GaCl}}$ with higher $p_{H_{2}}^{\circ }$ are significantly higher than those of $p_{Y}$, especially at lower *R*_Y_, which causes higher YN formation, specifically at a lower *R*_Y_. The *x*_Y_ vs. *R*_Y_ changes non-linearly with increasing $p_{H_{2}}^{\circ }$, which shows that even at very low *R*_Y_, a minimum *x*_Y_ ~0.1 can be obtained.

#### 3.2.3. V/III Effect on YGaN Deposition

**Figure 3:**
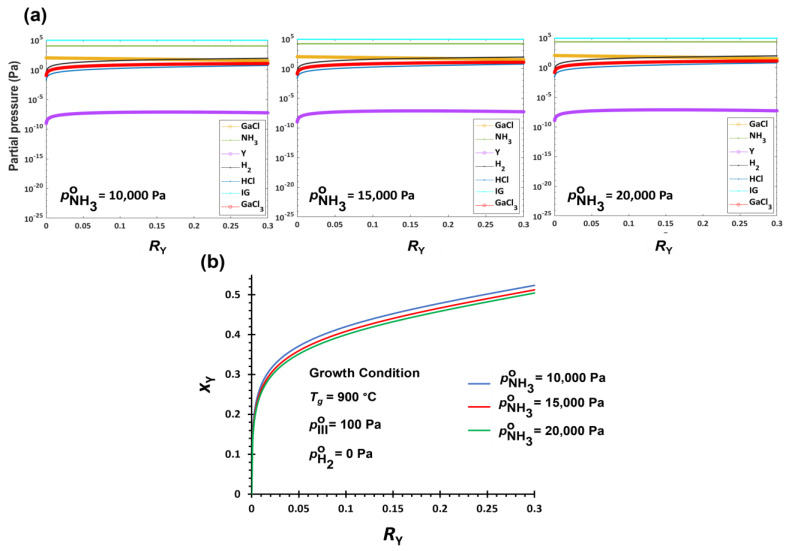
(**a**). Equilibrium partial pressures of carrier gas, reactants, and products in the growth zone of YGaN. (**b**). Mole fraction of YN, *x*_Y_ in the deposited Y*_x_*Ga_1−*x*_N solid film as a function of cation precursor input ratio, and R_Y_ with different NH_3_ input conditions (V/III ratios).

The V/III ratio is another critical growth parameter in the CVD method. Figure 3a shows the equilibrium partial pressure changes vs. *R*_Y_ at different V/III ratios in the range of 100–200 by changing $p_{{\mathrm{NH}}_{3}}^{\circ }$ = 10,000–20,000 Pa. The other conditions are fixed at *T_g_* = 900 °C, $p_{\mathrm{HCl}}^{\circ }$ = 0.000001 Pa, $p_{H_{2}}^{\circ }$ = 0 Pa, and $p_{\mathrm{III}}^{\circ }$ = 100 Pa. By increasing the V/III ratio, $p_{Y}$ and $p_{{\mathrm{GaCl}}_{3}}$ increase and $p_{\mathrm{GaCl}}$ decreases. The change in $p_{Y}$ is marginal as compared to the change in $p_{\mathrm{GaCl}}$. The driving force of GaN formation increases in the whole range of *R*_Y_, mainly because of $p_{\mathrm{GaCl}}$ reduction. Although the increase in $p_{{\mathrm{GaCl}}_{3}}$ with higher V/III ratio could reduce the GaN formation driving force, its effect on the *x*_Y_ is limited as compared to the decrease in $p_{\mathrm{GaCl}}$. Therefore, the composition of alloy becomes GaN-rich with the increasing V/III input ratio, as shown in Figure 3b. The change of *x*_Y_ in the whole range of *R*_Y_ shows the same trend at different V/III ratios. Also, the V/III ratio effect on the composition of YGaN alloy is less significant than the effects of hydrogen in the carrier gas.

#### 3.2.4. YAlN Deposition

Similar to Y*_x_*Ga_1−*x*_N, solid compositions (*x*_Y_) vs. cation precursor input ratios (*R*_Y_) are analyzed for Y*_x_*Al_1−*x*_N deposition. The driving force for AlN deposition is related to $p_{{\mathrm{AlCl}}_{3}}^{{}^{\circ}}-p_{{\mathrm{AlCl}}_{3}}$ determining the relative ratio of Al. The solid composition of Y, *x*_Y_ in Y*_x_*Al_1-*x*_N film is defined by the following equation:

(36) $$x_{Y}=\frac{p_{Y}^{{}^{\circ}}-p_{Y}}{\left(p_{Y}^{{}^{\circ}}-p_{Y}\right)+\left(p_{{\mathrm{AlCl}}_{3}}^{{}^{\circ}}-p_{{\mathrm{AlCl}}_{3}}\right)}$$

**Figure 4:**
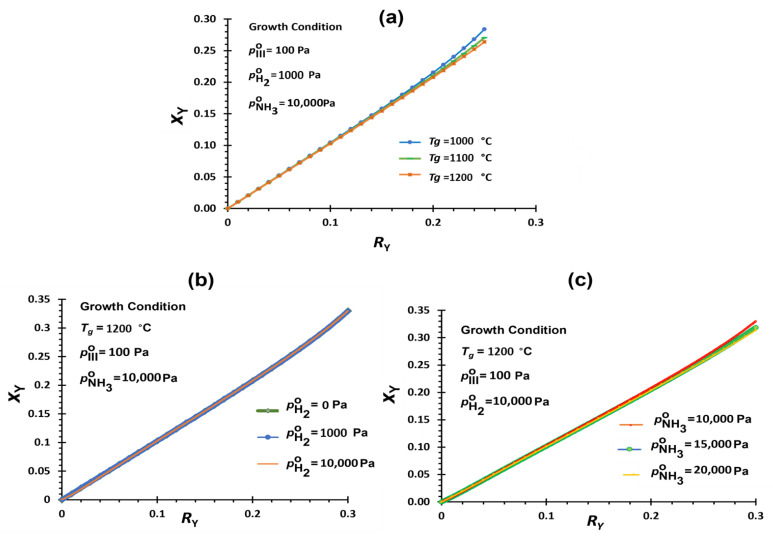
(**a**). Mole fraction of YN, *x*_Y_ in deposited Y*_x_*Al_1−*x*_N solid film as a function of cation precursor input ratio, *R*_Y_ at various growth temperatures, *T_g_*. (**b**). *x*_Y_ vs. *R*_Y_ with different H_2_ input conditions. (**c**). *x*_Y_ vs. *R*_Y_ with different NH_3_ input conditions (V/III ratios).

Equilibrium vapor pressures of a representative condition, e.g., *T_g_* = 1200 °C with $p_{H_{2}}^{\circ }$ = 0 Pa, $p_{{\mathrm{NH}}_{3}}^{\circ }$ = 10,000 Pa, and $p_{\mathrm{III}}^{\circ }$ = 100 Pa (V/III ratio = 100), are shown in Figure S8. Similar to the case of YGaN, $p_{Y}$ is the lowest among the species. However, $p_{{\mathrm{AlCl}}_{3}}$ in YAlN growth zone is lower than $p_{\mathrm{GaCl}}$ in YGaN growth zone by two orders of magnitude, suggesting stronger driving force of AlN deposition than that of GaN. As a result, the *x*_Y_ vs. *R*_Y_ relationship in YAlN is more linear than that in YGaN. Furthermore, growth temperatures (Figure 4a), hydrogen mixtures in the carrier gas (Figure 4b), and V/III ratios (Figure 4c) do not have significant effects on the *x*_Y_, unlike in the case of YGaN. While fewer substantial changes are observed by different growth conditions, their trends are different from those of YGaN. *x*_Y_ slightly decreases with higher *T_g_*. Increasing the hydrogen partial pressure does not affect the *x*_Y_ in YAlN. Table 1 summarizes the changes in *x*_Y_ of Y*_x_*Ga_1−*x*_N and Y*_x_*Al_1−*x*_N depending on different growth parameters. All three parameter changes of YAlN are similar to the ScAlN growth by HybCVD [25].

## 4. Discussion

### 4.1. Effect of Temperature and Hydrogen on YGaN Deposition

For the deposition of Y*_x_*Ga_1−*x*_N ternary alloys by HybCVD, the driving force of YN formation is significantly higher, and its changes with growth parameters are negligible. Therefore, *x*_Y_ is mostly determined by the change in the driving force of GaN formation, which is affected by the equilibrium pressures of both GaCl and GaCl_3_. The Gibbs free energies for the formation of GaN and GaCl_3_ become less negative at higher temperatures (Figure S5). At lower temperatures, e.g., *T_g_* < 700 °C, the Gibbs free energy of GaCl_3_ formation is more negative than that of GaN. Therefore, substantial amounts of the $p_{\mathrm{GaCl}}^{{}^{\circ}}$ are consumed for the formation of the by-product GaCl_3_ (Equation (20)). By increasing *T_g_* up to 1000 °C, the difference in the formation of GaCl_3_ and GaN becomes less, gaining higher driving force for the solid deposition; however, the driving force of GaN formation is still significantly lower than that of YN formation. Therefore, the solid composition of YGaN is heavily YN-rich even at very low Y precursor input, i.e., *x*_Y_ >> *R*_Y_. For example, *R*_Y_ = 0.002 at *T_g_* = 900 °C and V/III = 100 without H_2_ in the carrier gas is calculated to yield *x*_Y_ ≈ 0.2 (Figure S9). This discrepancy is mitigated by increasing *R*_Y_, i.e., a lesser amount of GaCl input. For example, *R*_Y_ = 0.3 results in the solid composition in the range of *x*_Y_ = ~0.4–0.5 (Figure 1b, Figure 2b and Figure 3b). As a result, the relationship of *x*_Y_ vs. *R*_Y_ in YGaN significantly deviates from the linearity. Furthermore, *x*_Y_ increases at higher temperatures as a result of decreasing the driving force of GaN formation while nearly the same driving force of YN formation is maintained, as described in Section 3.2.1.

Hydrogen is one of the by-products of the reactions in the growth zone (Equations (18)–(20)), which can reduce the driving force for the formation of both YN and GaN when $p_{H_{2}}^{\circ }$ increases. If the equilibrium constants (*K*) are the same, the effect of increasing $p_{H_{2}}^{}$ (the result of increasing $p_{H_{2}}^{\circ }$) is more considerable, i.e., more increase in $p_{Y}$ in Equation (21) than $p_{\mathrm{GaCl}}$ in Equation (22) because of the higher power of $p_{H_{2}}^{}$. However, *K*_6_ is much smaller than *K*_5_, thus being related to less negative Gibbs free energy (Figure S5). Therefore, the change in $p_{\mathrm{GaCl}}$ with a minor change in $p_{H_{2}}^{\circ }$ is more sensitive. The increase in $p_{\mathrm{GaCl}}$ (with higher $p_{H_{2}}^{}$) is in competition with the decrease in $p_{{\mathrm{GaCl}}_{3}}$ (Equation (23)). However, the effect of $p_{\mathrm{GaCl}}$ is more than $p_{{\mathrm{GaCl}}_{3}}$, which results in lower driving force for the GaN formation. Therefore, *x*_Y_ increases with more hydrogen in the carrier gas. At lower *R*_Y_, the increase in *x*_Y_ is more prominent. At the lower *R*_Y_, $p_{\mathrm{GaCl}}$ is more sensitive by increasing $p_{H_{2}}^{}$ because of relatively high *a*_GaN_, in addition to small *K*_6_ value. As a result, $p_{\mathrm{GaCl}}$ changed more significantly to compensate for the increase in $p_{H_{2}}^{\circ }$ which increases *x*_Y_ more considerably at lower *R*_Y_.

### 4.2. Comparison between YGaN and YAlN Deposition

The change of various deposition conditions shows different effects on the YGaN and YAlN compositions. The *x_Y_* vs. *R_Y_* does not follow the same trend for YGaN and YAlN. The dominant precursors of chloride in Al and Ga source zones are AlCl_3_ and GaCl, respectively, with different amounts of Cl content, which result in different reactions in the growth zone of YAlN and YGaN. The GaCl precursor involves an additional reaction in the growth zone of YGaN, producing GaCl_3_ and hydrogen by-products, and these are compared to YAlN, unlike AlCl_3_. *R_Y_*-*x_Y_* relationships in YGaN are non-linear in all the deposition conditions. In contrast, nearly linear *R_Y_*-*x_Y_* relationships are obtained in YAlN. Also, the presence of hydrogen as one of the by-products in the YGaN growth zone increases the *x_Y_* in Y*_x_*Ga_1−*x*_N, compared to *x_Y_* in Y*_x_*Al_1−*x*_N, by reducing the driving force of GaN formation more than that of AlN. An increase in hydrogen in the system has the most substantial effect on the increase in $p_{\mathrm{GaCl}}$ due to the very small amount of *K_6_* (Equation (22)). Also, by increasing the temperature, the Gibbs free energy formation of AlN becomes more negative, while it becomes less negative for GaN formation. Therefore, with higher *T_g_*, *x_Y_* increases in YGaN, while it decreases in YAlN.

A non-linear *R_Y_*-*x_Y_* relationship in YGaN, resulting in YN-rich alloy formation relative to the input cation precursor ratio, may pose a challenge in the composition control of the film. A small variation in *R_Y_* could cause a significant change in *x_Y_* (Figure S9). However, it is beneficial considering the huge difference in vapor pressures of GaCl and Y. The vapor pressure of Y in the Y source zone is lower than that of GaCl in the Ga-source zone by the order of four in magnitude, which requires very high-volume flow rates for carrier gas from the Y-source zone to the mixing zone to achieve similar amounts of Ga and Y precursors. It is not necessary to achieve similar amounts, as reasonable target composition for the enhanced piezoelectric properties, e.g., *x_Y_* = 0.1–0.2, can be achieved even with very limited input of Y precursor, e.g., *R_Y_* = 0.0005–0.002 (Figure S9). A nearly linear *R_Y_*-*x_Y_* relationship in YAlN requires similar amounts of Al and Y precursor inputs. The vapor pressure of AlCl_3_ in the Al-source zone (on the 10^−5^ atm [25]) is lower than that of GaCl in the Ga-source zone (~10^−4^ atm), hence the difference between Y and AlCl_3_ to be compensated is smaller.

## 5. Conclusions

In summary, thermodynamic calculations for the epitaxial growth and deposition of Y-alloyed GaN and AlN thin films by HybCVD, using precursors of chloride (GaCl and AlCl_3_), vapor-phase elemental source (Y) and hydride (NH_3_), showed the effects of common growth parameters in CVD on the compositions of Y in the films. For YGaN alloys, the relationship between the input cation precursor ratio, *R*_Y_, and composition, *x*_Y_, was not linear: the *x*_Y_ was always higher than *R*_Y_, indicating significantly higher incorporation of Y in the film. This characteristic is beneficial in achieving a target composition of the film for desired piezoelectric properties, even with a relatively small input precursor of Y originating from the low equilibrium vapor pressure of the precursor. Also, higher growth temperature (700 → 900 °C), more H_2_ in the carrier gas (0 → 100 Pa), and lower V/III ratio (200 → 100) resulted in higher *x*_Y_ by further decrease in driving force of GaN deposition. In contrast, *R*_Y_ vs. *x*_Y_ was nearly linear for YAlN alloys, and the effects of temperature, carrier gas mixture, and V/III ratio on *x*_Y_ were marginal. This study suggests that the HybCVD technique can be employed for the growth of piezoelectric Y-alloyed GaN and AlN materials.

## Figures and Tables

**Table 1:** Change of *x*_Y_ in Y*_x_*Ga_1−*x*_N and Y*_x_*Al_1−*x*_N by increase of growth temperature (*T_g_*), hydrogen (H_2_) carrier gas, and V/III input partial pressures.

	*T_g_* ↑	H_2_ ↑	V/III ↑
*x*_Y_ in Y*_x_*Ga_1−*x*_N	Increase	Increase	Decrease
*x*_Y_ in Y*_x_*Al_1−*x*_N	Marginal decrease	Nearly same	Marginal decrease

Upward arrow (↑) is an indication of increasing value of the parameters.

## Data Availability

Data presented in this article is available on request from the corresponding author.

## Supplementary Materials

The following supporting information is available at: https://www.mdpi.com/article/10.3390/nano12224053/s1, Figure S1: Gibbs free energy change of possible chemical reactions between Y and HCl for the formation of Y chlorides; Figure S2: Gibbs free energy change of a reaction between YCl_3_ and NH_3_ for the formation of YN; Figure S3: Equilibrium vapor pressure of Y over the condensed phase; Figure S4: Equilibrium partial pressures of gaseous species over Ga metal in the Ga source zone; Figure S5: Gibbs free energy change of reactions between Y and NH_3_ for the formation of YN, GaCl, and NH_3_ for the formation of GaN, GaCl, and HCl for the formation of GaCl_3_, AlCl_3_, NH_3_ for the formation of AlN, and AlCl_3_ for the formation of Al_2_Cl_6_; Figure S6: Logarithmic equilibrium constants for various reactions between Ga-chlorides and quartz; Figure S7: Gibbs free energy change of reactions between Y and possible source containers; Figure S8: Equilibrium partial pressures of reactants, products, and carrier gas in the growth zone of YAlN; Figure S9: Mole fraction of YN in deposited Y*_x_*Ga_1−*x*_N solid film with very small input cation precursor ratios.
