# Supplementary material for: Thermodynamic Analysis of Group-III-Nitride Alloying with Yttrium by Hybrid Chemical Vapor Deposition
Source: Nanomaterials (Basel). 2022 Nov 17;12(22):4053. doi: 10.3390/nano12224053 (PMC9698282; doi:10.3390/nano12224053)
Supplement: Supplementary file 1 [file nanomaterials-12-04053-s001.zip › nanomaterials-1907337-supplementary-done.pdf]

nanomaterials

Supporting Information

Thermodynamic Analysis of Group-III-Nitride Alloying with Yttrium by Hybrid Chemical Vapor Deposition

Mina Moradnia [1,2,3,†], Sara Pouladi [1,2,3,†], Muhammad Aqib [1,2,3] and Jae-Hyun Ryou [1,2,3,4,5,*]

[1] Department of Mechanical Engineering, University of Houston, Houston, TX 77204, USA
[2] Texas Center for Superconductivity at UH (TcSUH), University of Houston, Houston, TX 77204, USA
[3] Advanced Manufacturing Institute (AMI), University of Houston, Houston, TX 77204, USA
[4] Department of Electrical and Computer Engineering, University of Houston, Houston, TX 77204, USA
[5] Materials Science and Engineering Program, University of Houston, Houston, TX 77204, USA
* Correspondence: jryou@uh.edu
† These authors contributed equally to this work.

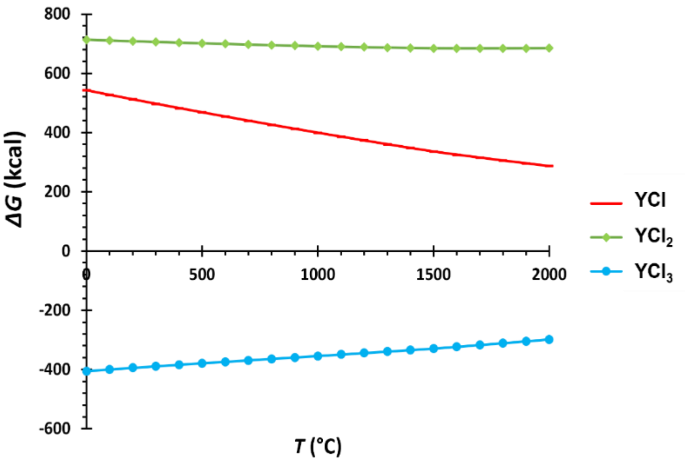

Figure S1. Gibbs free energy change (*ΔG*) as a function of temperature (*T*) of possible chemical reactions between Y and HCl for the formation of Y chlorides: YCl [Y (s,l) + HCl (g) → YCl (g) + 1/2H_2 (g)]; YCl_2 [Y (s,l) + 2HCl (g) → YCl_2 (g) + H_2 (g)]; and YCl_3 [Y (s,l) + 3HCl (g) → YCl_3 (g) +3/2H_2 (g)].

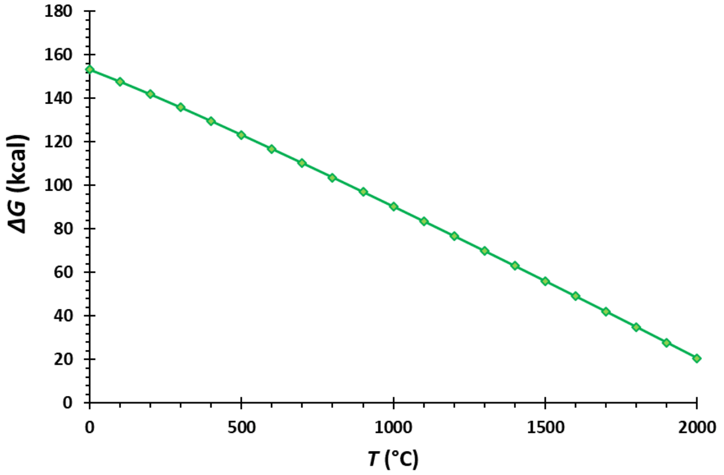

Figure S2. Gibbs free energy change (*ΔG*) as a function of temperature (*T*) of a reaction between YCl_3 and NH_3 for the formation of YN [YCl_3 (g) + NH_3 (g) → YN (s) + 3HCl (g)].

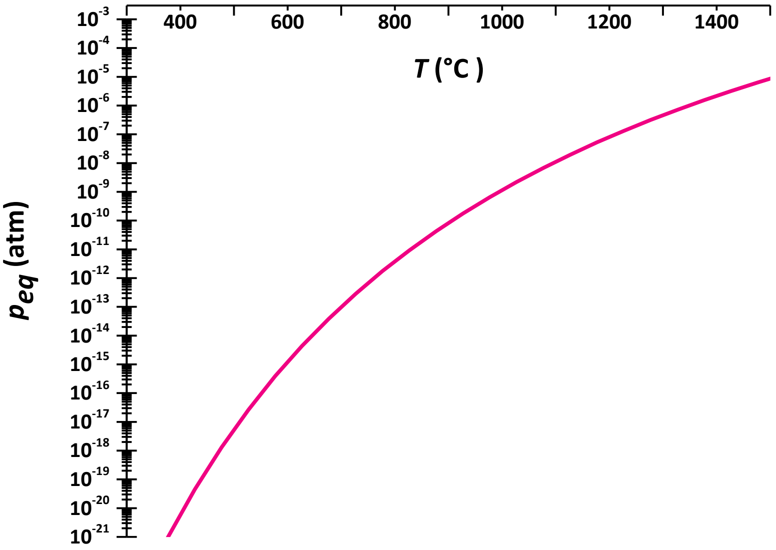

Figure S3. Equilibrium vapor pressure (p_{eq}) of Y over the condensed phase as a function of temperature (T).

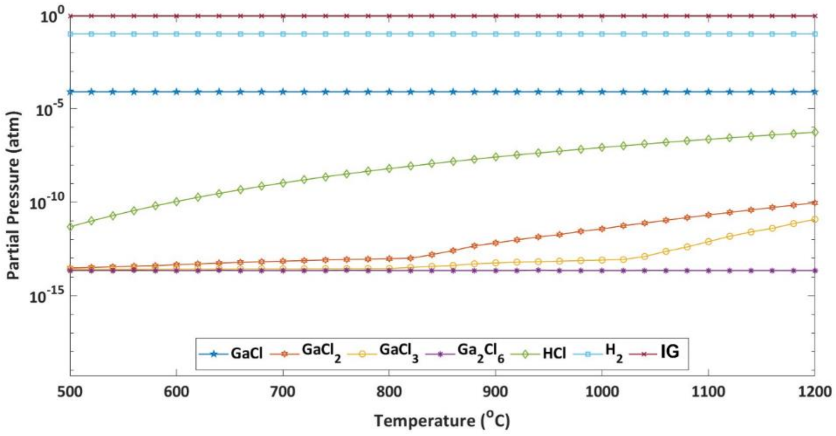

Figure S4. Equilibrium partial pressures (p_i) of gaseous species over Ga metal as a function of temperature (T) in the Ga source zone.

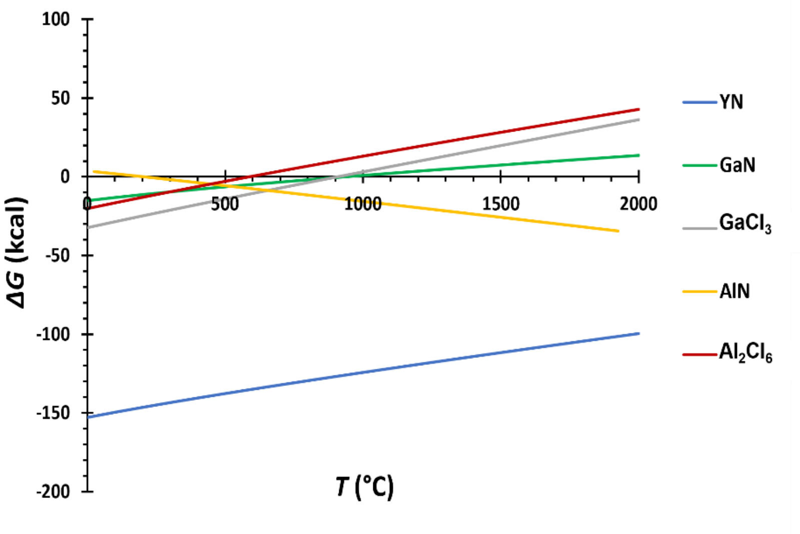

Figure S5. Gibbs free energy change (*ΔG*) as a function of temperature (*T*) of selected possible reactions in the mixing/growth zone: Y and NH_3 for the formation of YN [Y (g) +NH_3 (g) → YN (s) + 1.5H_2 (g)]; GaCl and NH_3 for the formation of GaN [GaCl (g) + NH_3 (g) → GaN (s) + HCl (g) +H_2 (g)]; GaCl and HCl for the formation of $GaCl_3$ [GaCl (g) + 2HCl (g) → $GaCl_3$ (g) + H_2 (g)]; $AlCl_3$ and NH_3 for the formation of AlN [$AlCl_3$ (g) + NH_3 (g) → AlN (s) + 3HCl (g)]; and $AlCl_3$ for the formation of Al_2Cl_6 [$AlCl_3$ (g) + $AlCl_3$ (g) → Al_2Cl_6 (g)].

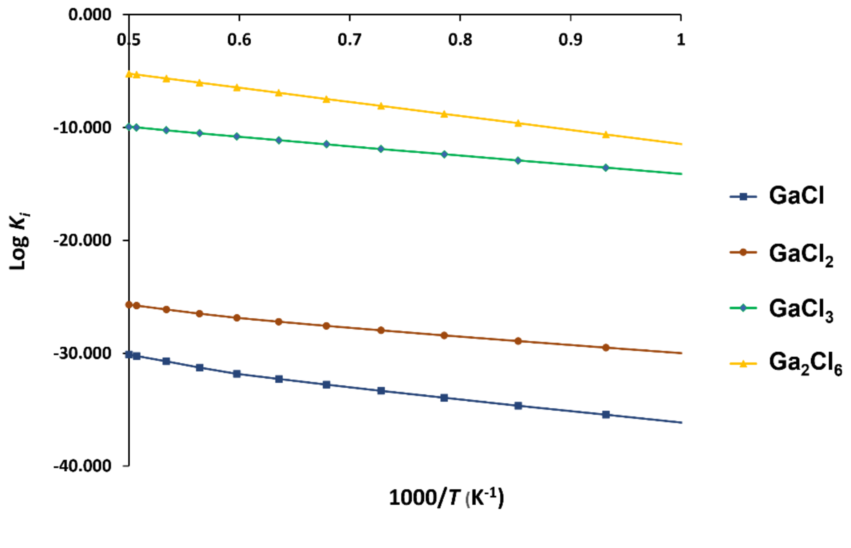

Figure S6. Logarithmic equilibrium constants (K_i) as a function of reciprocal temperature (1/*T*) for various reactions between Ga-chlorides and quartz (SiO_2): GaCl [4GaCl (g) + 3SiO_2 (s) → $SiCl_4$ (g) + 2Si (s) + 2Ga_2O_3 (s)]; $GaCl_2$ [4$GaCl_2$ (g) + 3SiO_2 (s) → 2$SiCl_4$ (g) + Si (s) + 2Ga_2O_3 (s)]; $GaCl_3$ [2$GaCl_3$ (g) + 1.5SiO_2 (s) → 1.5$SiCl_4$ (g) + Ga_2O_3 (s)]; and Ga_2Cl_6 [Ga_2Cl_6 (g) + 1.5SiO_2 (s) → 1.5$SiCl_4$ (g) + Ga_2O_3 (s)].

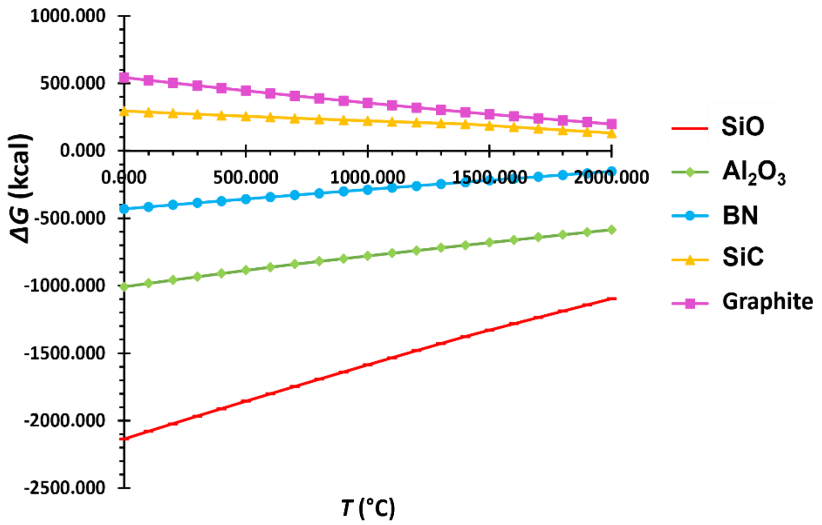

Figure S7. Gibbs free energy change (ΔG) as a function of temperature (T) of reactions between transition metal (Y) and possible source containers: SiO_2 [2Y (s) + 1.5SiO_2 (s) → Y_2O_3 (s) + 1.5Si (s)]; Al_2O_3 [2Y (s) + Al_2O_3 (s) → Y_2O_3 (s) + 2Al (s)]; BN [Y (s) + BN (s) → YN (s) + B (s)]; SiC [Y (s) + 2SiC (s) → YC_2 (g) + 2Si (s)]; and graphite [Y (s) + 2C (s) → YC_2 (g)].

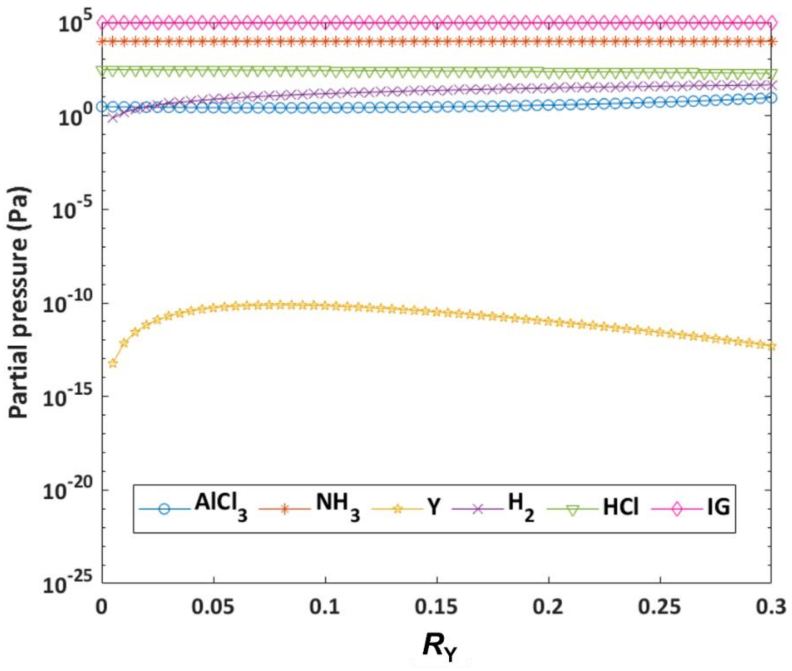

Figure S8. Equilibrium partial pressures of reactants, products, and carrier gas in the growth zone of YAlN as a function of cation precursor input ratio, R_Y at T_g = 1200 °C with $p^{\circ}_{H_2}$ = 0 Pa, $p^{\circ}_{NH_3}$ = 10000 Pa, and p°_{III} = 100 Pa (V/III ratio = 100).

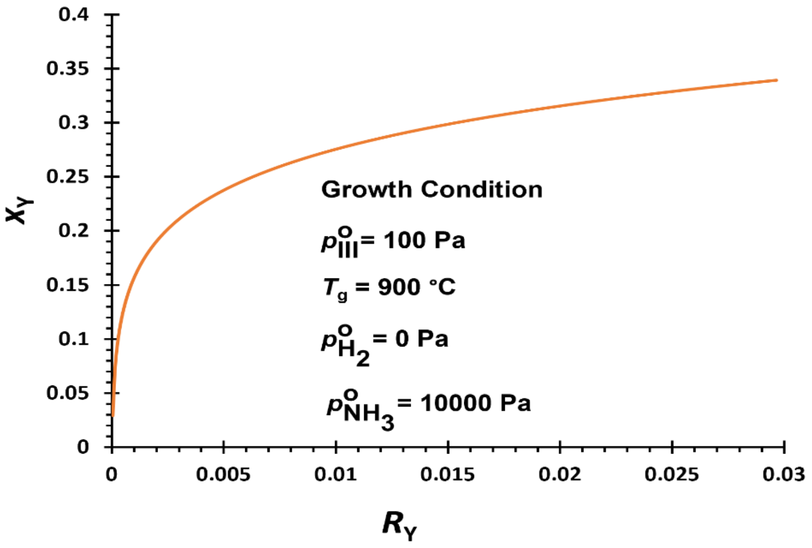

Figure S9. Mole fraction of YN, x_Y in deposited $Y_xGa_{1-x}N$ solid film at T_g = 900 °C as a function of cation precursor input ratio, R_Y less than 0.03.
